# Cigarette Smoke Induces Immune Responses to Vimentin in both, Arthritis-Susceptible and -Resistant Humanized Mice

**DOI:** 10.1371/journal.pone.0162341

**Published:** 2016-09-07

**Authors:** Mitali Bidkar, Robert Vassallo, David Luckey, Michele Smart, Kelly Mouapi, Veena Taneja

**Affiliations:** 1 Department of Immunology, Mayo Clinic, Rochester, Minnesota, United States of America; 2 Department of Internal Medicine, Division of Pulmonary and Critical Care Medicine, Mayo Clinic, Rochester, Minnesota, United States of America; 3 Division of Rheumatology, Mayo Clinic, Rochester, Minnesota, United States of America; H. Lee Moffitt Cancer Center & Research Institute, UNITED STATES

## Abstract

Rheumatoid arthritis (RA) is an autoimmune disease marked by chronic synovial inflammation and both, genetic and environmental factors are involved in its pathogenesis. Human leukocyte antigen (HLA) DRB1*0401 is associated with susceptibility to develop RA, while cigarette smoke (CS) exposure promotes seropositive disease with increased severity in DRB1*0401+ individuals. Smokers have higher levels of antibodies against citrullinated peptides. In this study, we determined whether the response to a known autoantigen, Vimentin (Vim) is shared epitope specific and how CS influences this response using transgenic-mice carrying RA-susceptible,*0401, and -resistant, *0402, genes. Following relatively brief exposure to CS, peptidyl arginine deiminase (PAD) enzyme expression was increased in murine lungs. Cigarette smoking led to production of Interferon (IFN)-γ with reduced levels of Interleukin (IL)-10 by splenocytes of *0401 mice. In contrast, CS augmented Th2 cytokines along with T-regulatory cells in *0402 mice. An increase in levels of antibodies to native and citrullinated Vim was observed in naïve mice of both strains following CS exposure. Our data showed that both arthritis-susceptible and -resistant mice can generate cellular and humoral immunity to Vim; however CS-induced modulation of host immunity is dependent on the interaction with the host *HLA* genes.

## Introduction

Rheumatoid arthritis (RA) is an autoimmune disease characterized by persistent synovial inflammation, marked by progressive destruction of articular cartilage and underlying bone, leading to disability [1]. It affects approximately 1% of world’s population and is characterized by presence of rheumatoid factor (RF) and anti-citrullinated protein antibodies (ACPA) in serum of affected individuals. It is a multifactorial disorder that involves an interplay between environmental and host genetic factors [2, 3]. A strong association between RA and human leukocyte antigen (*HLA*) DRB1*04 alleles has been suggested on the basis of the “shared epitope (SE)” hypothesis [4]. According to this hypothesis, shared sequences in the 3^rd^ hypervariable region comprising residues 67–74 among the alleles, DRB1*0401, *0404/0408 and *0405 are crucial for the selection of autoreactive T-cells pertinent to RA. On the other hand, known arthritis resistant *HLA*-alleles such as DRB1*0402, *0103, *1302,*0803 and *1102 do not positively select autoreactive cells [5, 6]. Among the environmental factors, cigarette smoke (CS) is robustly associated with predisposition to RA [7, 8]. Indeed, the risk for seropositive RA increases 21-fold in smokers carrying the *DR4* gene as compared with nonsmokers carrying no SE genes [9]. The effects of CS on the immune system are profound and complex; however the exact mechanism by which smoking modulates RA remains unknown. Several studies have suggested that oxidative stress, inflammation and enhanced cellular and humoral immunity in smokers may contribute to pathophysiology of RA [7, 10].

Citrullination is a post translational modification in which positively charged arginine residues are converted to neutral citrulline by a class of the calcium-dependent enzyme, peptidyl arginine deiminases (PAD). In an inflammatory environment, when apoptotic cells are inadequately cleared, their intracellular content (including citrullinated proteins and/or PAD enzymes) is released into the extracellular space. These citrullinated proteins are subsequently internalized, processed and presented by antigen-presenting cells (APCs), [11– 13]. PAD2 and PAD4 have been identified to be predominantly expressed in inflamed synovial tissue and citrullinate glycoproteins resulting in the production of ACPA [11, 14]. Vimentin (Vim), an intermediate filament, is widely expressed in macrophages and mesenchymal cells of synovium as well as in the lungs, making it a promising target of specific autoimmunity in RA [12]. Under normal physiological conditions, the Vim cytoskeleton is a highly dynamic structure undergoing polymerization and de-polymerization for various biological functions. In apoptotic cells however, citrullination of the non-α helical amino terminal head domain of Vim by PAD and further cleavage by caspases disrupts the Vim network causing it to collapse into perinuclear aggregates [15]. Antibodies to citrullinated-Vim (cit-Vim) are found in approximately 75% of RA patients and are associated with the presence of *DR4* [2]. In genetically susceptible individuals, CS plays an important role in ACPA production by inducing PAD expression to citrullinate lung proteins in bronchial macrophages [11].

In humans, it is challenging to define the role of a single *DR* gene to linkage due to disequilibrium with *DQ* alleles. Thus, *HLA* transgenic mice provide an opportunity to delineate the role of *HLA* genes in various immune responses [6]. Prior studies by our group showed that mice deficient for endogenous class II expression (AEo) and expressing functional *HLA DR*B1*0401 and *DQ8* transgenes develop collagen-induced arthritis (CIA) that mimics several features of human RA [16–18]. On the other hand, mice expressing DRB1*0402 are resistant to develop arthritis [19]. Our recent study using a humanized mouse model of arthritis suggested that interaction between *HLA* molecules and CS determines exacerbation of arthritis [8]. In the current study, we show that both arthritis-susceptible and -resistant mice can mount *exvivo* T cell proliferation to Vim. However in arthritis-susceptible mice, CS augments immunity with proinflammatory cytokine production, while arthritis-resistant mice are characterized by a Th2 profile with increased numbers of T-regulatory (T-reg) cells. Our study suggests that citrullination of Vim-epitopes upon CS exposure may lead to enhanced pro-inflammatory immune responses thus breaking tolerance in certain genetic backgrounds.

## Material and Methods

### Transgenic mice

The generation of transgenic (Tg) mice, DRB1*0401 and DRB1*0402 has been described previously [17]. All mice were on C57/B6 background and lacked all endogenous MHC class II chains, AE -/-. Female mice aged 8–10 weeks used in this study were bred and maintained in the pathogen-free Immunogenetics Mouse Colony at Mayo Clinic, Rochester, MN in accordance with the Animal Use and Care Committee. All the experiments and animal care were carried out with the approval of the Institutional Animal Use and Care Committee (IACUC). Animals were sacrificed by euthanasia in accordance with IACUC euthanasia guidelines. For convenience, DRB1*0401 and DRB1*0402 transgenic mice will be referred to as *0401 and *0402 respectively.

### Exposure to cigarette smoke

A manually controlled smoking chamber (Teague enterprises, CA, USA) was used to expose the mice to CS as previously described. The smoking chamber enabled exposure of the mice to a combination of mainstream and side-stream CS generated by 3R4F Kentucky research cigarettes [8]. Mouse strains, *0401 and *0402, were either allocated to cigarette Smoke (CS) exposure or were exposed only to environment air and used as controls (NS). Both of these groups were further challenged with recombinant-Vimentin (rVim) or else left unchallenged (naïve. Mice were exposed to regulated concentrations of CS generated from 2 cigarettes for 45 minute period, repeated 10 times a day for 5 days/week (Monday through Friday). Cigarette smoke-exposed mice were divided into 2 groups, naïve and primed. Naïve and primed mice were exposed to CS for duration of 5 weeks and the primed group received the antigen challenge in the final week of CS exposure. At the end of the experimental period, the spleens were removed and cells were harvested for *in vitro* T-cell proliferation, FACS and cytokine analysis. mRNA expression was studied using lung samples. Sera were collected at sacrifice.

### Flow cytometry

The cell surface expression of *HLA*-*DR* on the splenocytes was analyzed by flow cytometry using L227 (anti-*DR*) mAbs to characterize the presence of the transgene. Conjugated specific anti-CD4, CD25, CD11b, and CD19 mAbs were used to enumerate and analyze splenic cells by FACS. In accordance with the manufacturer’s instructions, the intracellular staining for FoxP3 was performed using Abs obtained from eBioscience and APC-conjugated rat IgG2a (BD Pharmingen) was used as the isotype control. All the markers were analyzed in triplicates and analysis was done using FlowJo software.

### Immunizations and T cell proliferation Assay

Transgenic mice (8–12 weeks old) were intradermally immunized with 200 μg of r-Vim (R&D Systems) emulsified 1:1 with Complete Freund’s Adjuvant (Difco Laboratories, Detroit, MI) at the base of the tail as previously described [17]. Splenic cells (1×10^6^) were cultured in HEPES-buffered RPMI 1640 containing 5% heat inactivated horse serum, streptomycin, and penicillin in 96-well round bottom tissue culture plates. Cells were challenged by adding 100μl of RPMI medium (negative control), concanavalin A (20 μg/ml, positive control), native and citrullinated-Vim (2.5μg/ml). Mice were also tested for T-cell reactivity to murine Vim-derived 20mer overlapping peptides. Native, citrullinated and Vim-derived peptides were synthesized and purified at Mayo Clinic Peptide Facility. The splenic cells were challenged *in vitro* with 100 μg/ml of each peptide and incubated for 48h at 37°C. The cultured cells were pulsed with ^3^H-thymidine (1μCi/well) during the last 18h. At the end of the assay, the cells were harvested using a plate harvester and incorporated radioactivity was determined using an automated counter (Microbeta, Perkin Elmer Wallac). Results were depicted as Stimulation Index (SI: ratio of mean cpm of triplicate culture to mean cpm of media). The SI value of 2 or more was taken as a positive response. Each experiment was performed using 2–3 mice per experiment/strain. Value for SI are provided in the text.

### Measurement of cytokines

Supernatants were collected from the splenocytes culture plates at the end of 72h and analyzed for interferon-γ (IFN-γ), interleukin-10 (IL-10), and IL-23, by ELISA using commercially available kits (BD Pharmingen).

### Measurement of anti-Vim (native and citrullinated) antibodies and rheumatoid factors (RFs)

Levels of anti-mouse Vim (native and citrullinated) IgG antibodies were measured in sera using a standard enzyme-linked immunosorbent assay (ELISA) as reported previously [8]. Rheumatoid factor, IgG and IgM levels were also measured in sera by ELISA as previously described [17]. Briefly, the ELISA plates were coated with rabbit IgG and kept at 4°C overnight. The plates were then washed, and serum in dilution of 1:40 was added and further incubated at room temperature for 45 mins. Subsequently, plates were washed with phosphate buffer saline containing 0.05% Tween 20 and incubated with horseradish peroxidase-conjugated rabbit anti-mouse IgG (Fc specific) or anti-mouse IgM (μ-chain specific) for 1h (both from Pierce, Rockford, IL). Plates were then washed again, added with 3,3’,5,5’-tetramethylbenzidine substrate (Sigma, St. Louis, MO). The absorbance spectrum was determined with an automated spectrophotometer (Bio-Rad, Hercules, CA). All assays were performed in duplicate and the data was reported as optical density (OD).

### Real-time PCR

Levels of mRNA of PAD2, PAD4 and cytokines were analyzed using real-time polymerase chain reaction (RT-PCR). RNAeasy columns (Qiagen) were used to extract RNA from cells and then cDNA was prepared using RNase H-reverse transcriptase (Invitrogen). Gene expression was analyzed in duplicates using the SYBR Green ERqPCR Super Mix kit (Invitrogen, USA). The expression level of each gene was quantified using the threshold cycle (Ct) method normalized for the housekeeping gene GAPDH and ß-actin.

### Statistical analysis

The comparison between the groups for T cell proliferation, antibody levels, cytokines and surface markers was done using non-parametric Mann-Whitney test. *p* ≤0.05 was as considered significant.

### Ethics Statement

All the experiments and animal care were carried out with the approval of the Institutional Animal Use and Care Committee (IACUC). Animals were monitored daily to make sure they were able to get food and water and did not lose more than 10% of weight. Animals were sacrificed by euthanasia in accordance with IACUC euthanasia guidelines at the termination of the experiments or if they lost more than 10% body weight or were unable to get water and food.

## Results

### PAD expression increases in the lungs of mice exposed to CS

**Fig 1:**
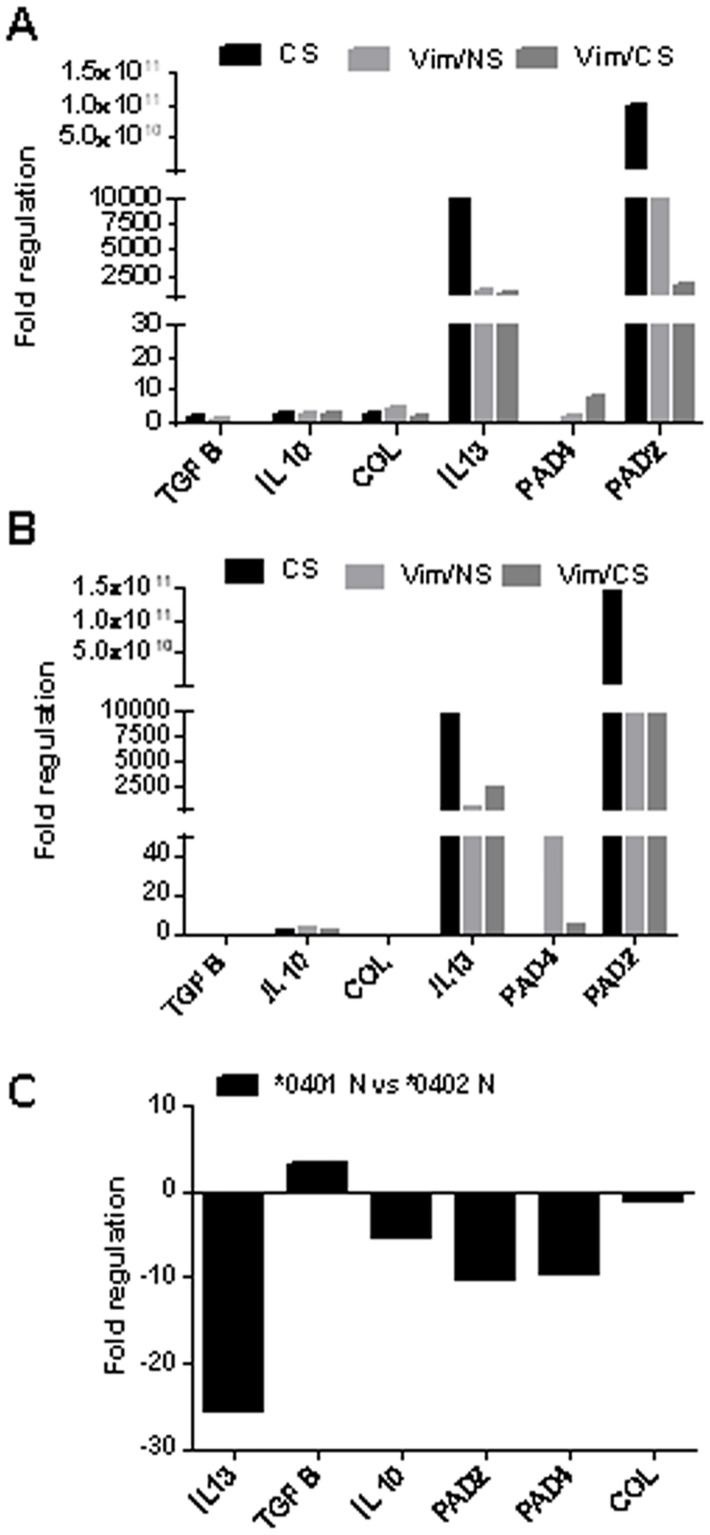
CS augments expression of PAD enzymes. The levels of mRNA transcripts for PAD 2/PAD4 enzymes and cytokines (IL-13 and TGF-β) were quantified in the lungs of naïve mice exposed to CS and, Vim-immunized mice before (Vim/NS) and after exposure to CS (Vim/CS), (A) *0401 mice and (B) *0402 mice. Naïve mice of both strains were used as control. (C) The m-RNA expression of PAD enzymes and cytokines in naïve *0401 mice in comparison with naïve *0402. The expression levels of each gene was calculated using ΔΔC_t_ method by normalizing C_t_ for the housekeeping gene GAPDH and ß-actin and is expressed in terms of fold regulation. N = 3-4mice/ strain. CS- cigarette smoke, NS-control “non smoker”.

Cigarette smoking increases lung PAD enzymes expression and, as PAD2 and PAD4 isotypes are important candidates to citrullinate Vim. We investigated whether CS modulates expression of these enzymes in arthritis-susceptible *0401 and–resistant *0402 mice. The mRNA transcript levels of PAD enzymes and cytokines (IL-13 and TGFβ) in *0401 (Fig 1A) and *0402 (Fig 1B) mice exposed to various conditions was analyzed by gene expression analysis of lung tissue. The PAD2 gene expression was elevated in both *0401 and *0402 naïve mice following CS exposure. Further, this expression was found to be higher in *0402 than in *0401 mice. Interestingly, CS exposed naïve mice showed higher PAD2 expression compared with the antigen-primed mice implying that, CS alone is a sufficient and strong inducer of PAD2 gene expression in this murine model. In arthritis resistant 0402 mice, CS exposure was associated with decreased PAD4 gene expression following Vim immunization, whereas in the susceptible 0401 mice, CS augmented PAD4 gene expression following Vim immunization. Exposure of naïve mice to CS up-regulated the expression of IL-13 in lungs of both strains (Fig 1A and 1B). Interestingly, CS exposed Vim-immunized *0402 mice had higher IL-13 mRNA transcripts in comparison with *0401 (Fig 1A and 1B). Further, a higher expression of TGF-β was observed in lung cells of *0402 naïve mice as compared with *0401 mice (Fig 1C).

### CS modulates the T-cell response to Vim epitopes in mice with RA-susceptible and non-susceptible HLA genes

**Fig 2:**
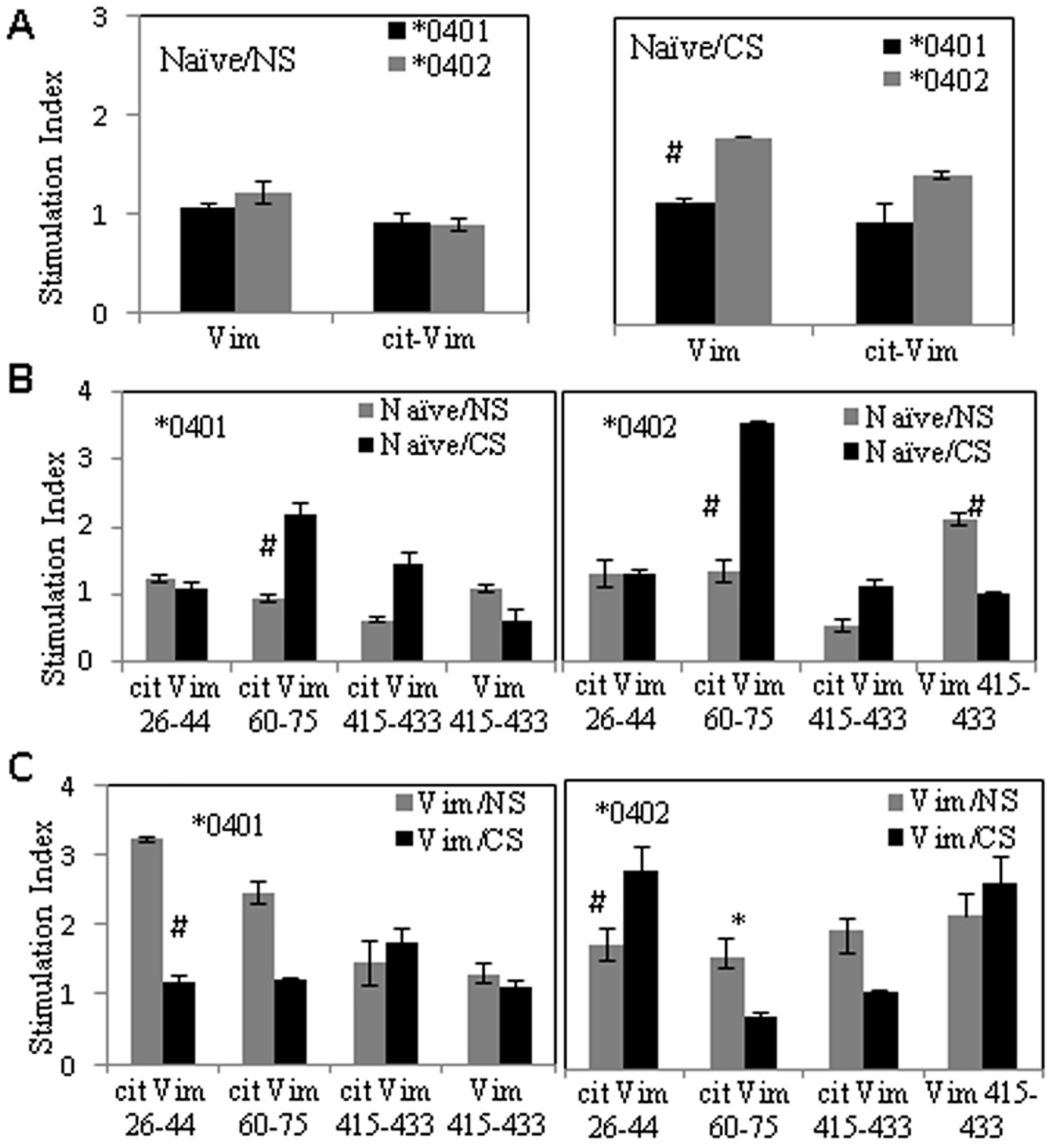
Cigarette Smoke modulates antigen-specific T-cell response in naive and Vim-primed mice. In vitro T-cell response to (A) native/citrullinated Vim, and (B) Vim-derived peptides before (NS) and after exposure to CS in naïve *0401 and *0402 mice. (C) T-cell proliferation to Vim-derived peptides in Vim-immunized mice exposed to CS or control (NS). CS-cigarette smoke, NS-non smoker. N = 2-3mice/ group. * p = 0.04; #p = 0.08.

In the next experiments, we sought to determine whether the adaptive immune response to Vim is influenced by host MHC II alleles and if CS impacts that response. To test this, we measured *ex vivo* antigen–specific T-cell proliferation in splenocytes of naïve and Vim-immunized mice exposed to CS and air-exposed mice (non-smoker controls). Cells were challenged *in vitro* to native and citrullinated Vim as well as, Vim-derived peptides in naïve and Vim-primed conditions, before and after exposure to CS (Fig 2). Following CS exposure, splenocytes from naïve *0402 mice proliferated to native Vim though the differences were not statistically significant (p = 0.08) (Fig 2A). Earlier studies have identified immunodominant Vim epitopes recognized by RA patients and healthy individuals [20–23]. Our current results are concordant with those studies, though both strains generated response to different epitopes. While all the 20mers peptides from Vim were studied, only the ones with differences in the trend between the 2 strains are shown here. The splenocytes from CS exposed naïve *0401 and *0402 mice generated a higher T-cell proliferation to cit-Vim 60–75 (NS vs CS, *0401; mean S.I. = 1 and 2.2 respectively, and *0402 S.I = 1.3 and 3.5 respectively; p = 0.08 for both strains). Interestingly, naïve *0402 mice mounted a higher T-cell response to Vim 415–430 which was suppressed by exposure to CS (Fig 2B) (NS vs CS, S.I = 1 and 2.5 respectively, p = 0.08. Immunization with Vim led to *ex vivo* T cell proliferation to Cit-Vim 26–44 in *0401 mice which was suppressed by CS exposure (Vim/NS vs. Vim/CS, mean S.I. = 3.2, and 1.1, p = 0.08). On the other hand, Vim-immunized *0402 mice showed an opposite trend eliciting a robust T-cell proliferation to cit-Vim 26–44 after CS exposure, (Vim/NS vs. Vim/CS, mean S.I. = 1.7 and 2.7 respectively, p = 0.08) while response to cit-Vim 60–75 was significantly suppressed (p = 0.04). Interestingly, T-cell response to Vim 415–433 after Vim immunization was only generated by *0402 mice, both before and after CS exposure (S.I. = 2.1 and 2.5 respectively) (Fig 2C). This suggests that both strains can generate response to various epitopes of Vim which is modulated by CS.

### CS promotes differential cytokine profile in DRB1*0401 and DRB1*0402 mice

**Fig 3:**
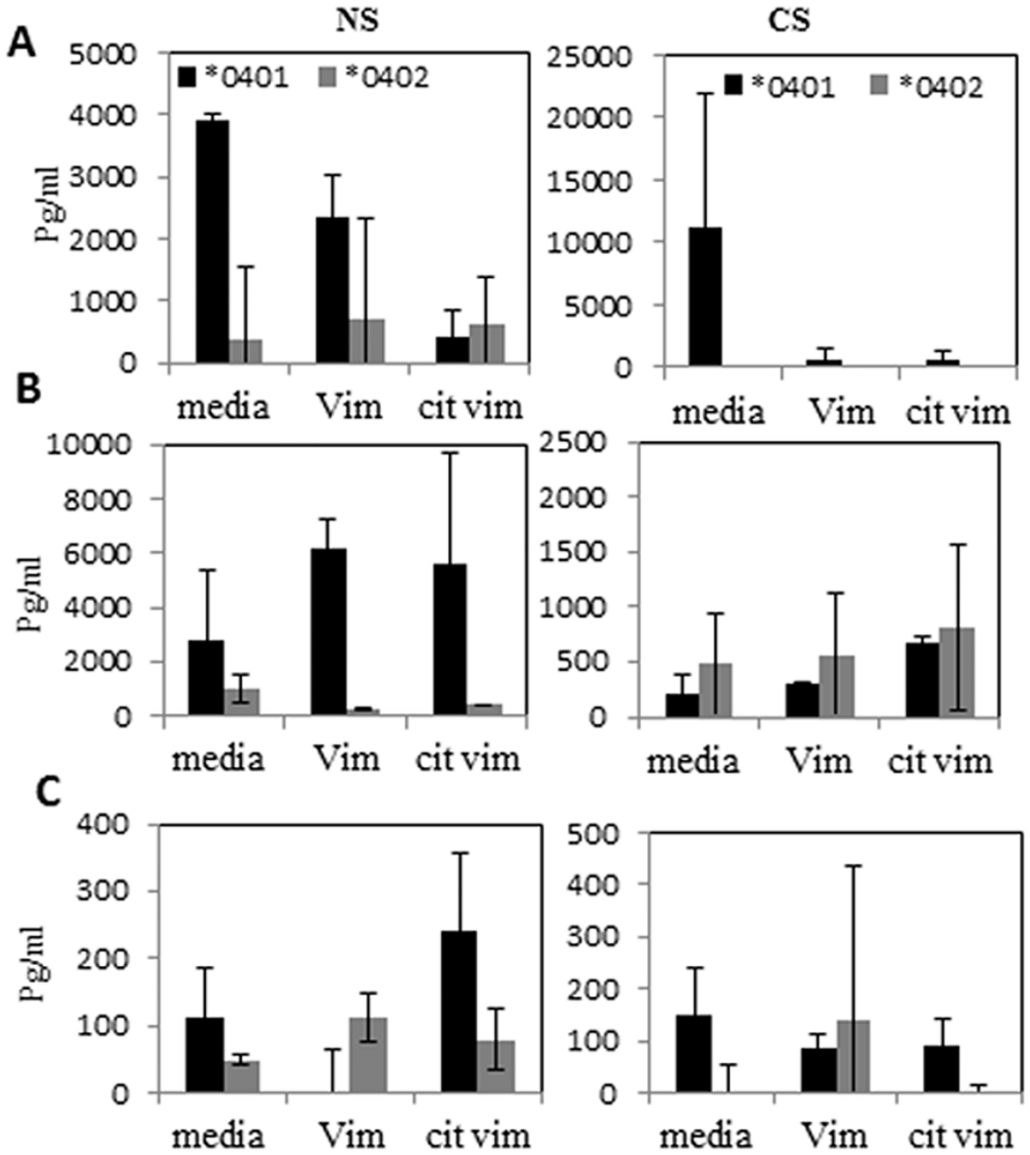
Differential effect of CS on cytokine production in Vim-primed *0401 and *0402 mice. (A) IFN-γ, (B) IL-10,and (C) IL-23produced in vitro culture of splenocytes in the presence of Vim or cit-Vim were measured. NS-non-smoker, CS- cigarette smoke.

Since CS modulated T cell response, we next determined whether CS modulates the cytokine profile to Vim differentially in arthritis–susceptible and–resistant mice. Cytokines [IFN-γ (Th1), IL-23 (Th17) and IL-10 (Th2)] produced *ex vivo* by splenocytes to native and citrullinated Vim were measured (Fig 3). A trend towards higher levels of IFN-γ and lower levels IL-10 production was observed from splenocytes of Vim-primed and CS exposed *0401 mice as compared to *0402 mice (Fig 3A and 3B). In contrast, n exposure to CS led to an augmentation of IL-10 production in Vim-primed *0402 mice as compared with *0401 mice compared to a higher IL-10 production by *0401 NS mice, differences were not significant (Fig 3B). CS exposure did not influence IL-23 production in *0402 mice, whereas in *0401 mice it led to reduced levels (Fig 3C). This data imply that CS differentially affects cytokine production in *0401 and *0402 mice.

### CS augments antigen-specific humoral responses

**Fig 4:**
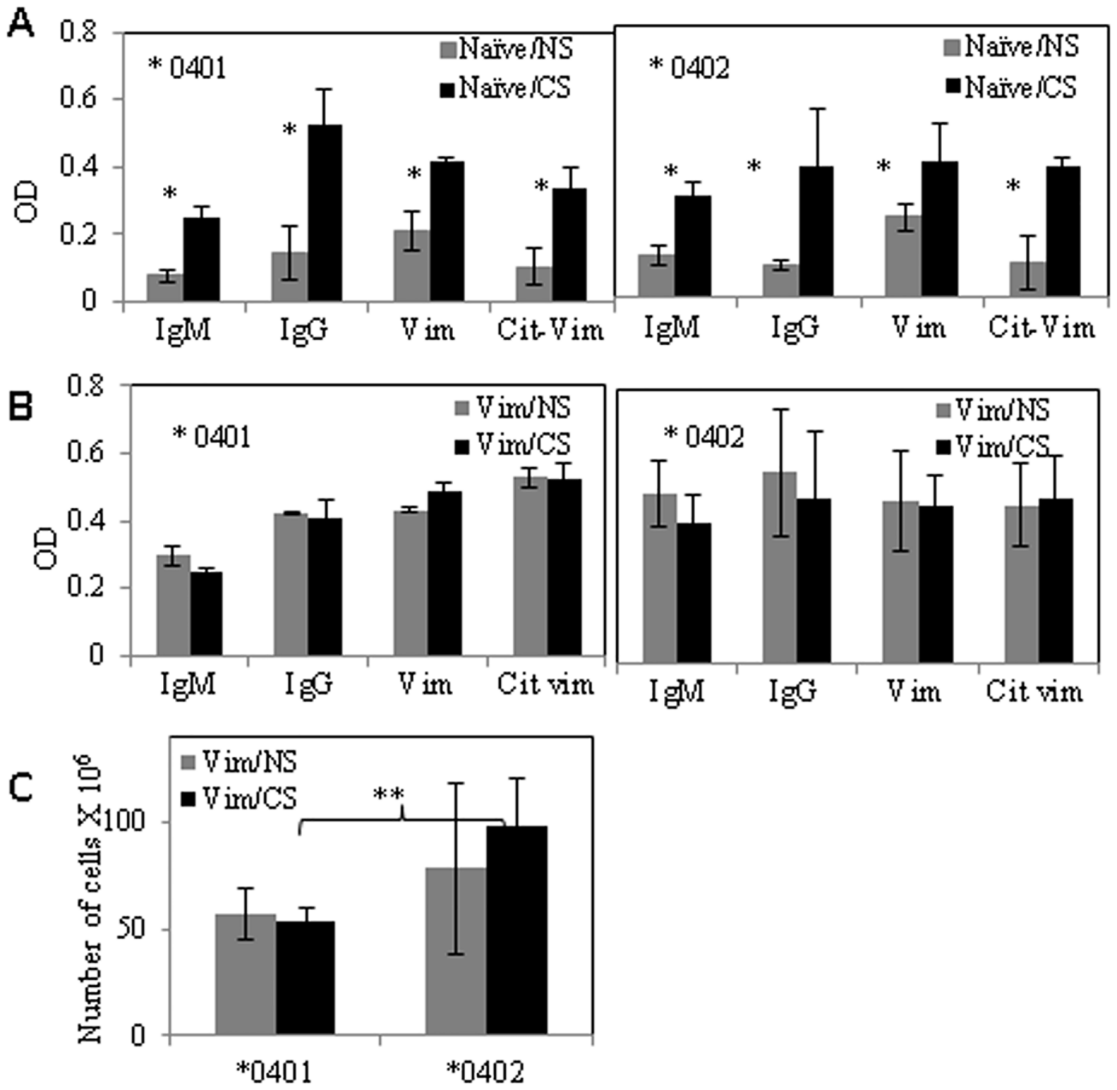
CS exposure augments antibody production in naïve mice in both strains. (A) Naïve mice showed an overall antibody augmentation upon CS exposure in both strains. (B) Immunization with Vim augmented antibody production in both strains. (C) Histogram shows the numbers of CD19^+^splenic B-cells (mean ± SD) from mice primed with Vim. Smoking induces proliferation of CD 19^+^ splenic B-cells in *0402 mice. *,p ≤0.05; **,p ≤ 0.009, *0401 vs *0402. N = 3/group.

Since CS is associated with seropositive RA, we evaluated if CS exposure changes antibody production in naïve mice and mice immunized with Vim. Rheumatoid factors (RF -IgG and -IgM) as well as auto-antibodies against native and citrullinated Vim were measured in sera of mice (Fig 4). Cigarette smoke exposure significantly augmented antibody levels in naïve mice of both strains. In *0401 mice, IgM (P = 0.05-), IgG (p = 0.05), anti-Vim (p = 0.05) and anti-Cit-Vim (p = 0.05) antibodies were increased significantly after exposure to CS. CS exposure led to a significant increase in all antibodies except to native Vim protein in *0402 mice (IgM, p = 0.03; IgG, p = 0.03 and anti-Cit-Vim p = 0.03) (Fig 4A). Immunization with Vim increased humoral response in both strains resulting in an increase in all antibodies in both strains, though CS did not modulate antibody levels in Vim-immunized mice (Fig 4B). Since CS enhanced antibody levels, we determined if CS also influenced B-cell numbers in *0401 and *0402 mice. No difference was observed in B cell numbers between *0401 mice NS and CS mice [5.7±1.2 10^7^ and 5.4±0.5 ×10^7^ in NS and CS respectively, n = 3/group]. CS exposure did not increase B-cell numbers in *0402 mice [7.8±4.0×10^7^ and 9.8±2.2×10^7^ in NS and CS respectively, n = 3/group, p = ns], although it was significantly higher relative to *0401 mice (Fig 4C, p = 0.009). However, both strains had similar antibody levels in Vim-immunized mice suggesting B cells may be activated in *0401 mice.

### CS leads to expansion of regulatory T-cells in *0402 but not DRB1*0401mice

**Fig 5:**
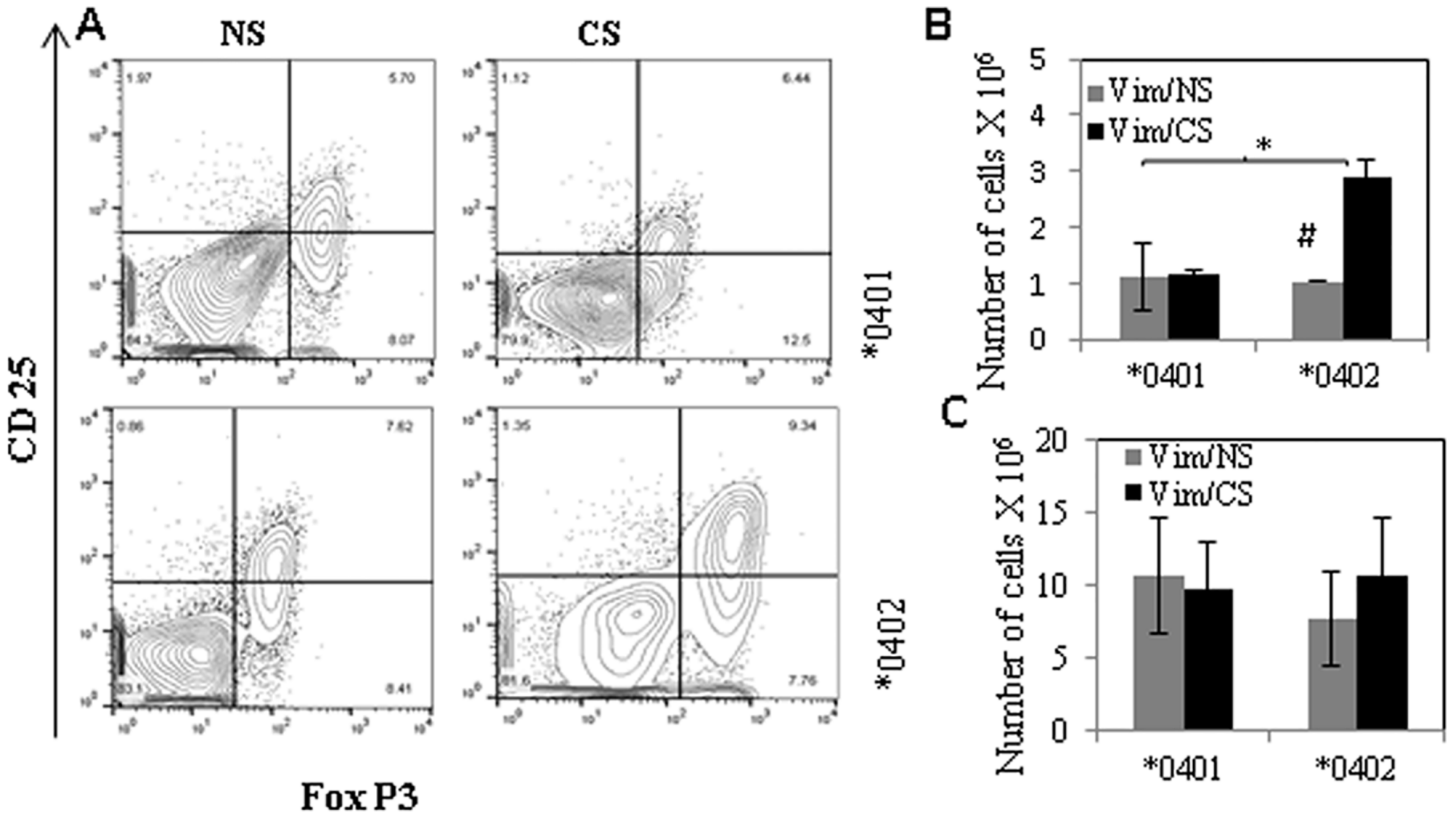
T-regulatory cell numbers—increase in *0402 mice after exposure to CS. Splenic cells from Vim primed mice were isolated and total numbers of CD4^+^ cells expressing CD25^+^FOX P3^+^ were enumerated by FACS using specific conjugated antibodies, before and after exposure to CS. (A) Dot plots showing CD25^+^ FOX P3 ^+^ cells from CD4^+^ gated cell population (B) absolute numbers (mean ± SD) of T-reg cells in Vim primed mice before and after exposure to CS. (C) Histogram shows the absolute numbers of CD 11b^+^ splenic cells (mean ± SD) in Vim primed mice with exposure to CS (Vim/CS) and control (Vim/NS).*,p = 0.04, #p = 0.08.

We have previously shown that *0402 mice harbor higher T-reg cells in comparison to *0401 mice [19]. Here we determined the potential mechanisms by which *0402 confers resistance to arthritis despite evidence of *ex-vivo* augmentation of adaptive antigen-specific immunity after CS exposure. Impact of CS on T-reg cells was determined by enumerating absolute numbers of CD4+CD25+FoxP3+T-reg cells from splenocytes harvested from Vim-immunized mice (Fig 5). Interestingly, CS exposure did not influence T-reg cells in *0401 mice, but led to an increase in the numbers of CD4+CD25+FoxP3+T-reg cells in *0402 mice (Fig 5A and 5B, *0402 vs *0401Vim/CS p = 0.04, *0402 NS vs CS p = 0.08).

Further, as CS is known to modulate immunity via its effects on antigen presenting cells, we tested whether it differentially affects splenic macrophage numbers in *0401 and *0402 mice. Absolute numbers of CD11b+ cells in splenocytes were enumerated before and after exposure to CS. When compared with NS mice, CS exposed *0402 mice demonstrated an increase in number of CD11b+ cells, although this did not achieve statistical significance, P>0.5 [0.7±0.3× 107 and 1±0.4×107in NS and CS mice respectively, n = 4] (Fig 5C).

## Discussion

An impressive body of epidemiologic and genetic research has shed light on the critical role played by gene-environment interaction in the etiology of RA [24]. Cigarette smoke has been studied extensively for its association with seropositive RA in genetically susceptible individuals. We have previously shown that CS exacerbates arthritis in DQ8 mice [8]. In this study we have shown that both RA-susceptible or RA-resistant alleles generate response to Vim though genotype influences response following CS.

Cigarette smoke has been associated with modulating immunity by influencing the expression of PAD enzymes [1, 8, 25]. The current data suggests that CS leads to increased PAD2 enzyme expression in both strains, suggesting that while RA-susceptible *0401 individuals may be more likely to have higher citrullinated peptides, this may not be solely explained by CS exposure. PAD enzymes are primarily present in monocytes (PAD4) and macrophages (PAD2 and PAD4) [15]. PAD2-expressing macrophages are increased in broncho-alveolar lavage (BAL) fluid from smokers relative to non-smoker control subjects [26]. We observed a systemic increase in number of macrophages upon exposure to CS in *0402 mice. Interestingly, the expression of PAD4 was increased in CS-exposed *0401 mice after immunization with Vimentin. While an association between CS and higher antibody production has been suggested in *0401+ RA patients [27], *0402+ subjects have not been studied. Our study suggests that CS impacts expression of PAD enzymes in both strains in an allele specific manner.

Cigarette smoke is associated with an increase in cellular and humoral responses to Cit-Vim in 75% of RA patients [2]. T-cells reactive to cit Vim-derived peptides have been reported in *DR*4 positive RA patient [20, 22]. The present observations are in line with the previous studies suggesting that CS can generate autoreactivity [2]. However, here we show that CS augmented antigen-specific T-cell responses to native and citrullinated Vim in naïve humanized mice carrying RA- susceptible and-resistant genes. Our observations are consistent with prior published data demonstrating the presence of Cit-Vim reactive CD4+ T-cells in both, *0401+ RA-patients as well as healthy controls [22, 23], suggesting that the presence of antigen-specific T-cells is insufficient to drive RA development. The lungs have been suggested as an initiating site for RA-related autoimmunity, particularly in cigarette smokers, in whom antibody levels to citrullinated proteins are elevated compared to non-smokers. [2]. We observed an overall increase in antibody response upon exposure to CS, with augmentation of anti-native and cit-Vim antibodies and RF. Anti cit-Vim antibodies, found in 20–47% of RA patients, have been suggested to play a role in the initiation or progression of rheumatoid synovitis [28]. Interestingly when primed with Vim, arthritis-resistant *0402 mice showed a much higher total antibody levels, with higher titers of IgM as compared to arthritis-susceptible *0401 mice on exposure to CS.

Our results suggest that both strains can generate antigen-specific response to various epitopes of Vim which is modulated by CS. Upon exposure to CS, both strains showed reactivity to Cit-Vim 60–75, an epitope to which reactive T-cells are present in RA patients [22]. Antibodies to Cit-Vim 60–75 are associated with early changes and increased destruction of bone and higher levels of serum receptor activator of nuclear factor kappa B ligand (RANKL) in ACPA-positive individuals before disease onset [21], implying that adaptive immunity directed to this epitope may be pathogenic. In that regards, only *0401 mice generated reactivity to cit-Vim 60–75 and cit-Vim 26–44 upon immunization with Vim. On the other hand, *0402 mice mounted T-cell response to native Vim 415–433, a peptide known to be naturally processed epitopes of Vim [20]. There is some evidence that these epitopes can be recognized by T-cells with a memory phenotype in patients with RA [20].

Activated T-cells can mediate pathogenic autoimmunity by activating macrophages via IFN-γ-dependent pathways or secretion of soluble macrophage-activating factors. These activated macrophages may secrete inflammatory proteins, which can perpetuate and amplify the inflammatory process [29, 30]. We observed an increased production of IFN-γ in Vim-immunized *0401 mice after exposure to CS. On the other hand, CS exposed *0402 mice produced higher levels of IL-10 which is known to play a protective role in RA by maintaining homeostasis of the immune system [31]. IL-10 is also reported to reduce *HLA*-*DR* expression and antigen presentation in macrophages and monocytes, while inhibiting the production of pro-inflammatory cytokines that are involved in the differentiation of Th1 response [32]. Further, Vim-immunized *0402 mice showed an increase in the number of B-cells upon CS exposure as compared to *0401 mice. Mouse B-cells may also contribute to IL-10 production in *0402 mice which is supported by the higher titers of Th2 cell-dependent IgM antibodies in *0402 mice. This data suggests that even though CS augments cellular response to similar epitopes, it differs in specificity between the arthritis-susceptible and–resistant strains.

The influence of genetic factors on CS-dependent effects on immunity was further evident by the observation of increased T-reg cells in CS exposed Vim-immunized*0402 mice. T-reg cells have been suggested to play a vital role in suppressing aberrant and excessive immune response for maintaining homeostasis [33]. While the exact significance of this is unknown in the present study, an increase in Treg cells and polarization of immune response to Th2 in *0402 mice provide an explanation for why *0402 mice exposed to CS do not develop arthritis (unpublished results). T-reg cells suppress inflammation by a variety of mechanisms and are an ideal target for remission in treatment of RA [34]. Compelling evidence has now demonstrated that TGF-β is important in the regulation of immune system by influencing T-reg cell function and in the differentiation of Th17 cell from CD4+ T-cells [35]. The intraperitoneal administration of TGF–β1 has been shown to reduce the incidence and severity of arthritis in an animal model [36]. We observed a higher expression of TGF-β was observed in *0402 as compared to *0401 mice. In addition, IL-13 was also augmented upon exposure to CS in both alleles, however the expression was higher in Vim-immunized *0402 as compared to *0401 mice. IL-13 is a pleiotropic cytokine that can act anti- or pro-inflammatory [37, 38]. The ability of IL-13 to decrease the production of proinflammatory cytokines by macrophages suggests a protective role in RA [37, 39].

## Conclusion

In summary, while Vim is considered as an autoantigen in RA, both *0401 and *0402 molecules can present many of the Vim-derived peptides to which patients harbor reactive T-cells, suggesting reactivity to Vim may not be the sole cause of initiation of RA. Host genotype interaction with smoking governs immune response in an allele-specific manner. Our findings suggest that CS modulates that response differentially in both strains resulting in different cytokine milieu which may be a key factor in determining the outcome of autoimmune disease.
